# Supplementary figures and images for: The retrieval of previously learned motor memories is facilitated by the reinstatement of default mode network manifold structures
Source: PLoS Biol. 2026 Mar 10;24(3):e3003684. doi: 10.1371/journal.pbio.3003684 (PMC12974888; doi:10.1371/journal.pbio.3003684)

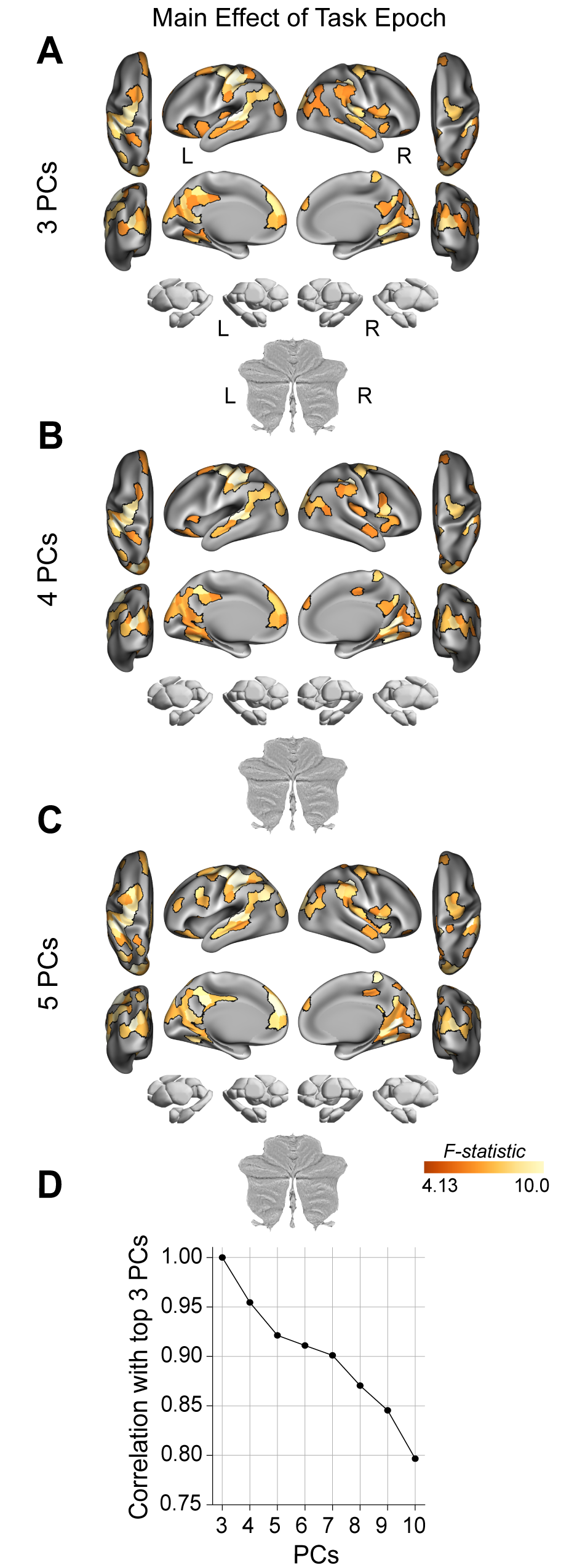

Supplement: S1 Fig — (A–C) Brain maps showing the main effect of Task Epoch on manifold eccentricity, calculated using the top 3 PCs (A), top 4 PCs (B), and top 5 PCs (C), respectively. The results show a high degree of correspondence in the significant effects across the different numbers of PCs. (D) Effect of including lower variance-explained PCs on the main effect brain maps. The plot shows the spatial correlation between the main effect map from our primary analysis (using 3 PCs) and maps generated using a progressively larger number of PCs (from 4 to 10). Note the high degree of spatial correlation, even as additional PCs are added to the data. (TIF) [file pbio.3003684.s001.tif]

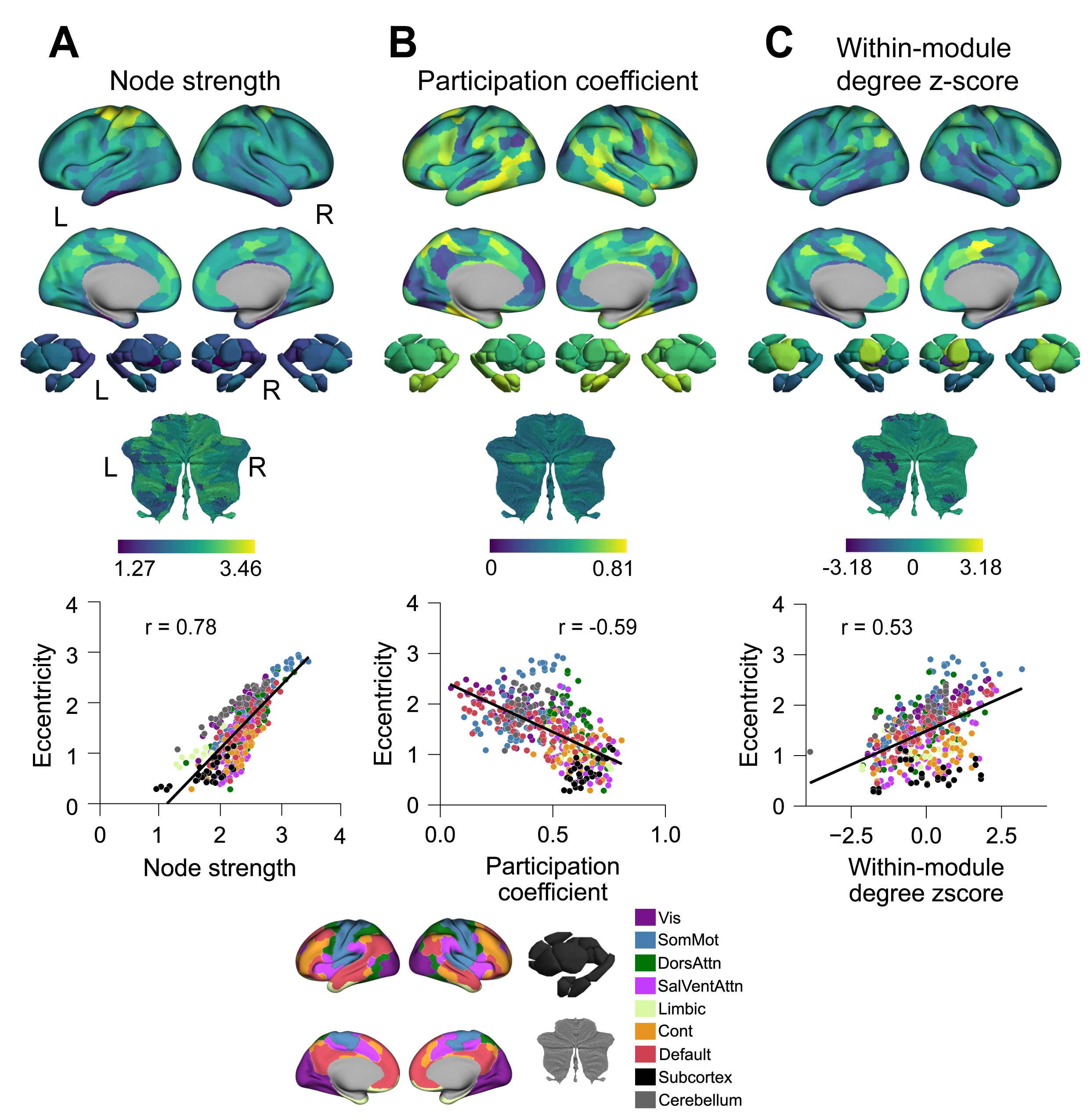

Supplement: S2 Fig — For three different graph theoretical measures—node strength (A), participation coefficient (B), and within-module degree z-score (C)—the top panels show the spatial map of each measure derived from the group-average Baseline connectivity matrix. The bottom panels show the corresponding scatterplot relating each measure to regional eccentricity. We found that Baseline eccentricity was positively correlated with node strength (r = 0.78, p < 0.001), positively correlated with within-module degree z-score (r = 0.53, p < 0.001), and negatively correlated with the participation coefficient, a measure of cross-network integration (r = −0.59, p < 0.001). Together, these results support the idea that areas with higher eccentricity generally have stronger functional coupling with other parts of the same functional network (i.e., higher segregation) whereas areas with lower eccentricity generally have stronger connectivity across different networks (i.e., higher integration). (TIF) [file pbio.3003684.s002.tif]

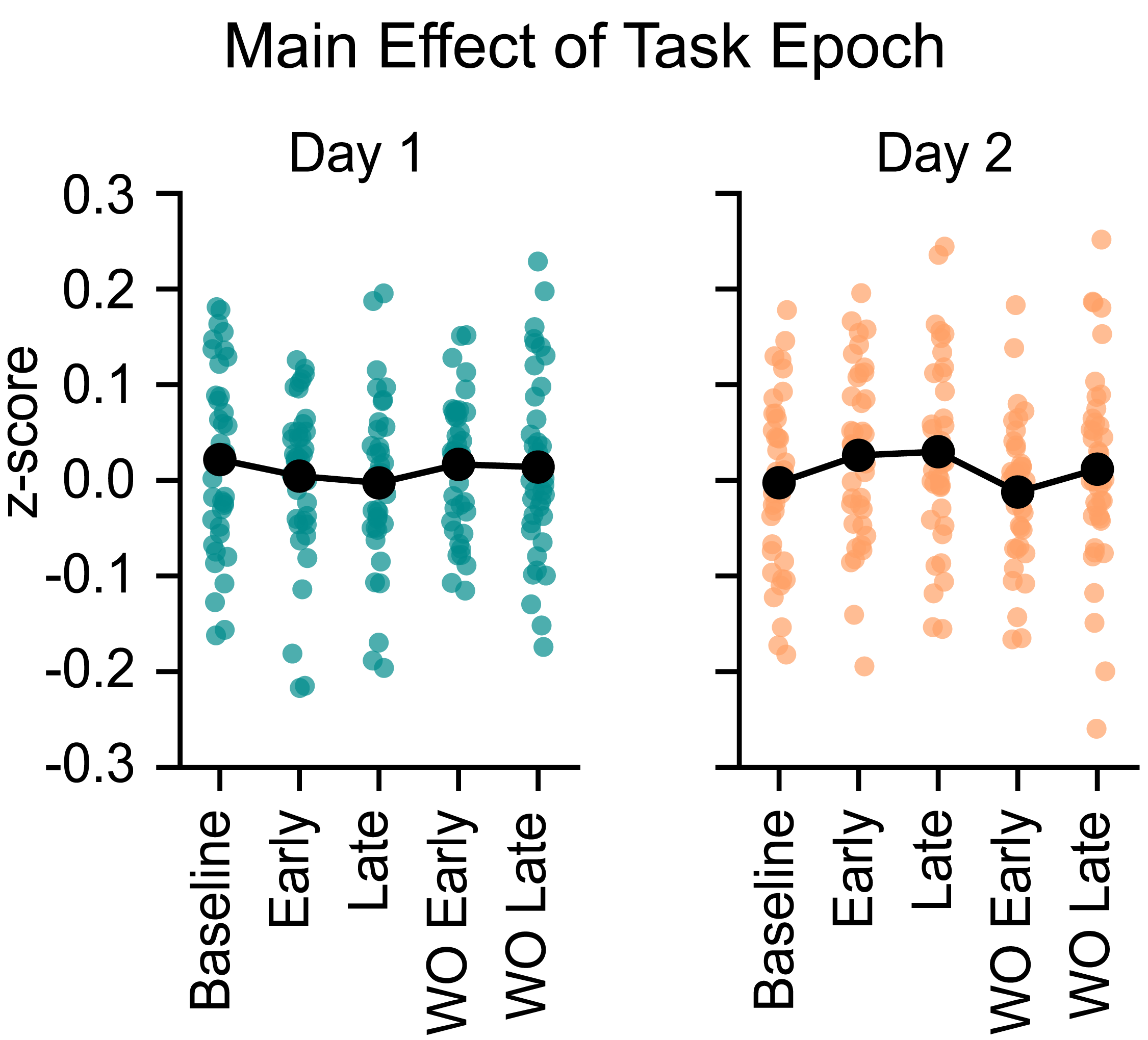

Supplement: S3 Fig — The plots show the mean BOLD activation (z-scored time series data, averaged across participants) for each task epoch within the regions that exhibited a significant main effect of Task Epoch in our primary connectivity analysis. The line overlay shows the group average across these regions, color-coded by day (Day 1: cyan; Day 2: orange). A two-way repeated measures ANOVA (Day x Task Epoch) on this activation data revealed no significant effects in any region after FDR correction (q < 0.05). This null result indicates that the manifold eccentricity changes described in the main text are not driven by simple changes in mean BOLD amplitude. The plots show mean BOLD activation (within-epoch mean of z-scored time series) for each task epoch within regions that exhibited a significant main effect of Task Epoch in our primary connectivity analysis. Underlying numerical data are provided in S1 Data and archived on Zenodo (https://doi.org/10.5281/zenodo.18613054). (TIF) [file pbio.3003684.s003.tif]

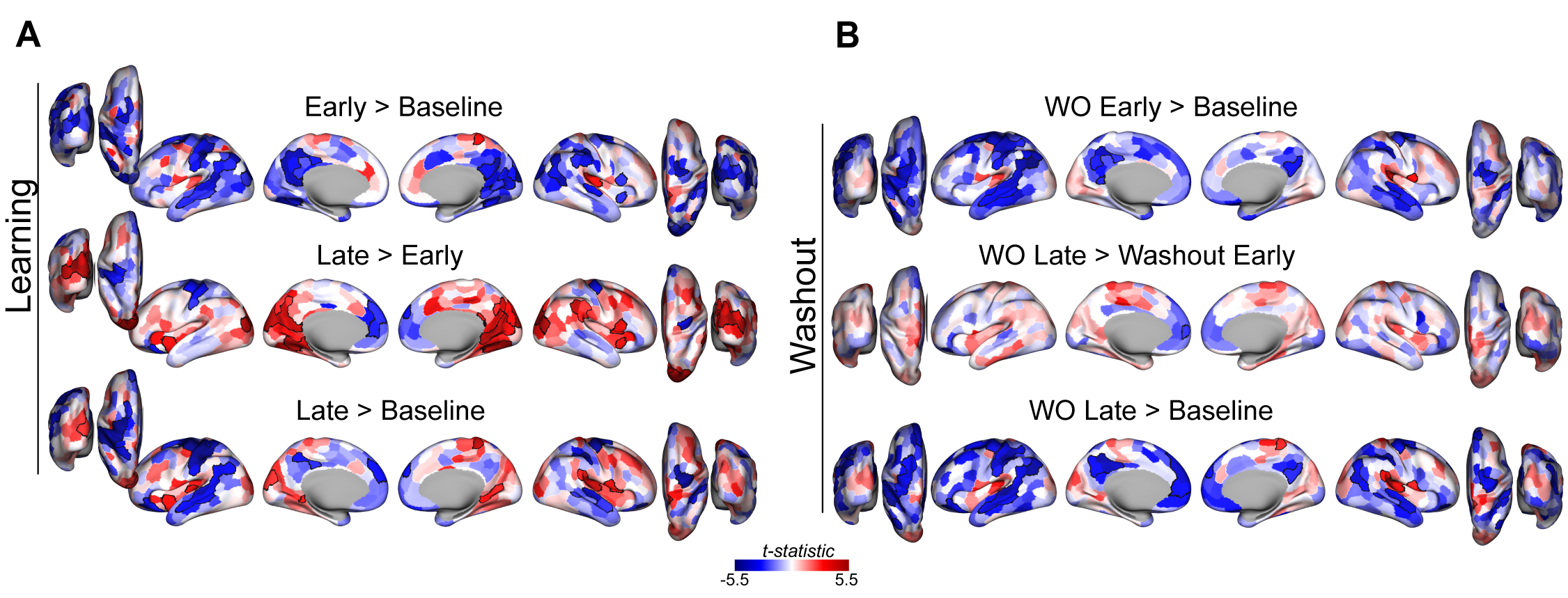

Supplement: S4 Fig — This figure shows the unthresholded statistical maps from the pairwise t-tests presented in Fig 3E. The maps compare eccentricity between task epochs, averaged across Day 1 and Day 2. Red indicates expansion (increased eccentricity), while blue indicates contraction (decreased eccentricity). (TIF) [file pbio.3003684.s004.tif]

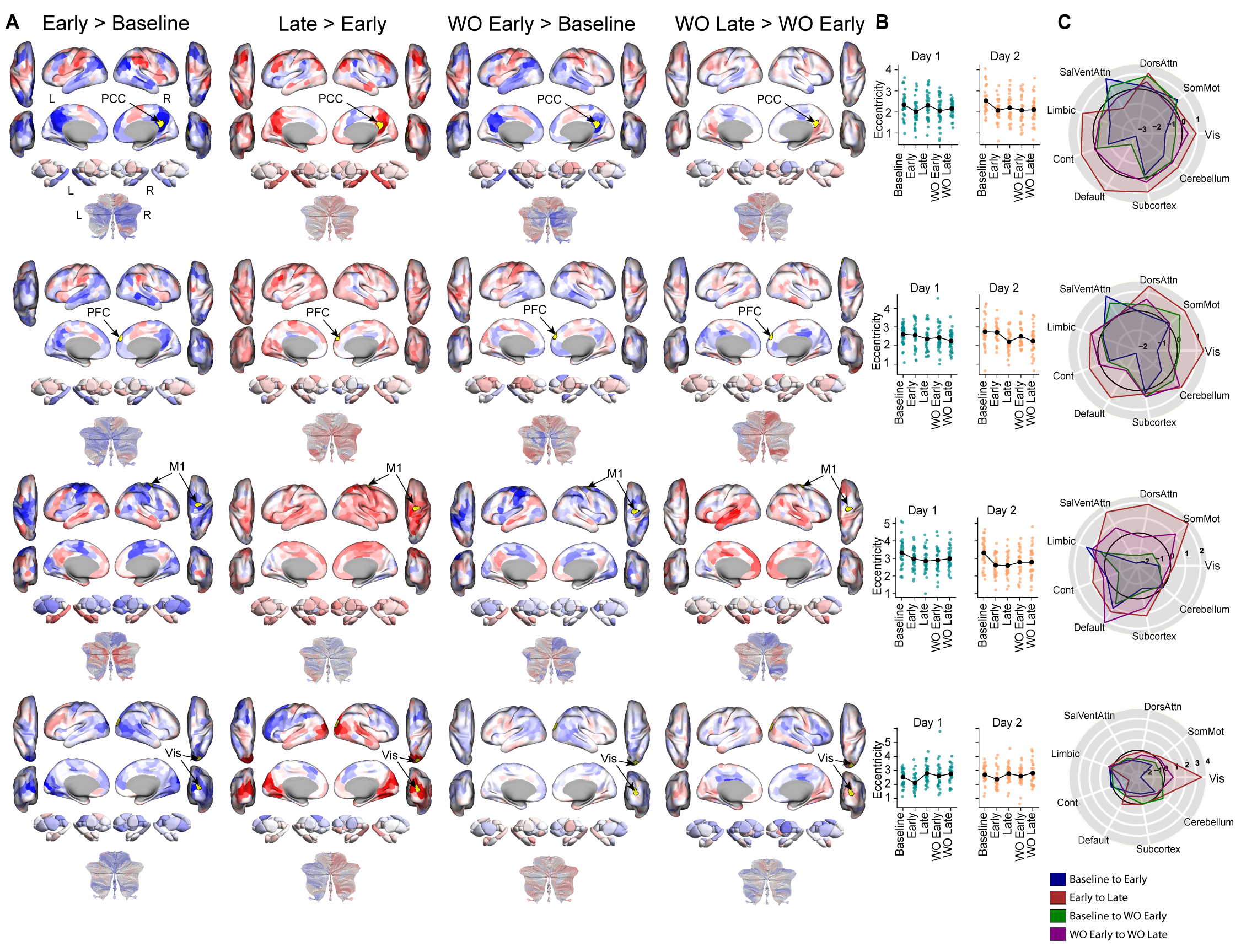

Supplement: S5 Fig — This figure illustrates changes in whole-brain functional connectivity for representative seed regions (indicated in yellow and by arrows) selected based on the significant main effect of Task Epoch from the ANOVA (see Fig 3A). Each row corresponds to a specific seed region. (A) Brain maps display the results of paired t-tests contrasting connectivity patterns between different task epochs (averaged across Day 1 and Day 2). For a given contrast (e.g., Early > Baseline) red denotes increased connectivity in the first epoch relative to the second, while blue denotes decreased connectivity. Specific contrasts are shown above the maps. (B) Scatter plots show the manifold eccentricity trajectory for the corresponding seed region across all 10 task epochs. Individual points represent participant means per epoch, color-coded by Day (Day 1: cyan, Day 2: orange). The solid line overlay indicates the group mean, illustrating the consistency of eccentricity changes across both days. (C) Spider plots summarize the network-level profile of the connectivity changes shown in panel (A), aggregated according to the Yeo 7-network parcellation (77). The radial axis represents the t-statistic for the change in connectivity between the seed region and each target network for the specified contrast (e.g., Early > Baseline). Points outside the black circle (t = 0; no change) indicate an increase in connectivity for that contrast, while points inside the circle indicate a decrease. Underlying numerical data are provided in S1 Data and archived on Zenodo (https://doi.org/10.5281/zenodo.18613054). (TIF) [file pbio.3003684.s005.tif]

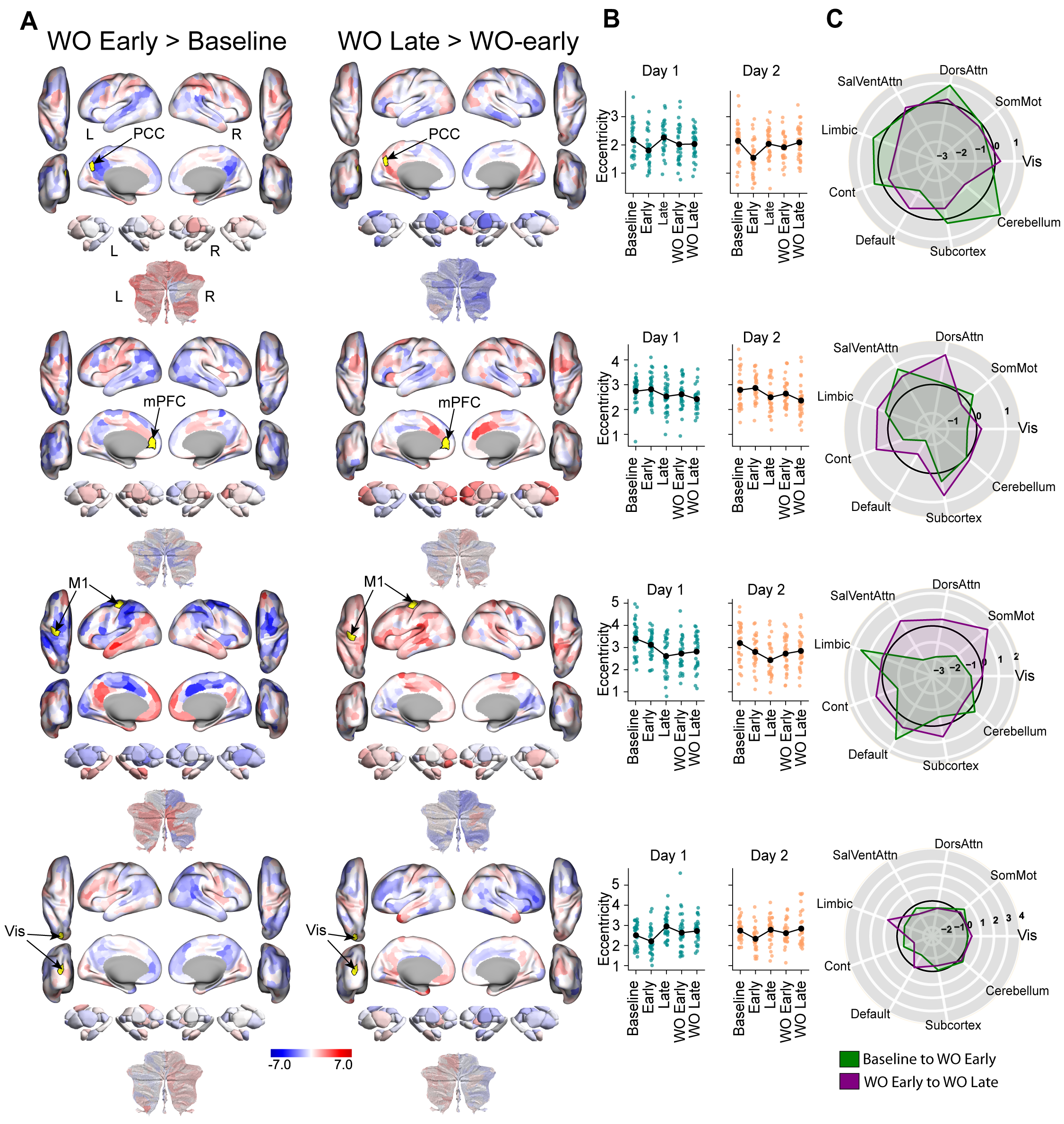

Supplement: S6 Fig — This figure illustrates changes in whole-brain functional connectivity for representative seed regions (indicated in yellow and by arrows) selected based on the significant main effect of Task Epoch from the ANOVA (same regions from Fig 5). Each row corresponds to a specific seed region. (A) Brain maps display the results of paired t-tests contrasting connectivity patterns between different washout task epochs (averaged across Day 1 and Day 2). For a given contrast (e.g., WO-early > Baseline), red denotes increases in connectivity in the first epoch relative to the second, while blue denotes decreased connectivity. Specific contrasts are shown above the maps. Note that the connectivity changes observed across the seed regions resemble those seen during learning (Fig 5), though they are comparatively more subtle. (B) Scatter plots show the manifold eccentricity trajectory for the corresponding seed region across all 10 task epochs. Individual points represent participant means per epoch, color-coded by Day (Day 1: cyan, Day 2: orange). The solid line overlay indicates the group mean, illustrating the consistency of eccentricity changes across both days. (C) Spider plots summarize the network-level profile of the connectivity changes shown in panel A, aggregated according to the Yeo 7-network parcellation (77). The radial axis represents the t-statistic for the change in connectivity between the seed region and each target network for the specified contrast (e.g., WO-early > Baseline). Points outside the black circle (t = 0; no change) indicate an increase in connectivity for that contrast, while points inside the circle indicate a decrease. Underlying numerical data are provided in S1 Data and archived on Zenodo (https://doi.org/10.5281/zenodo.18613054). (TIF) [file pbio.3003684.s006.tif]

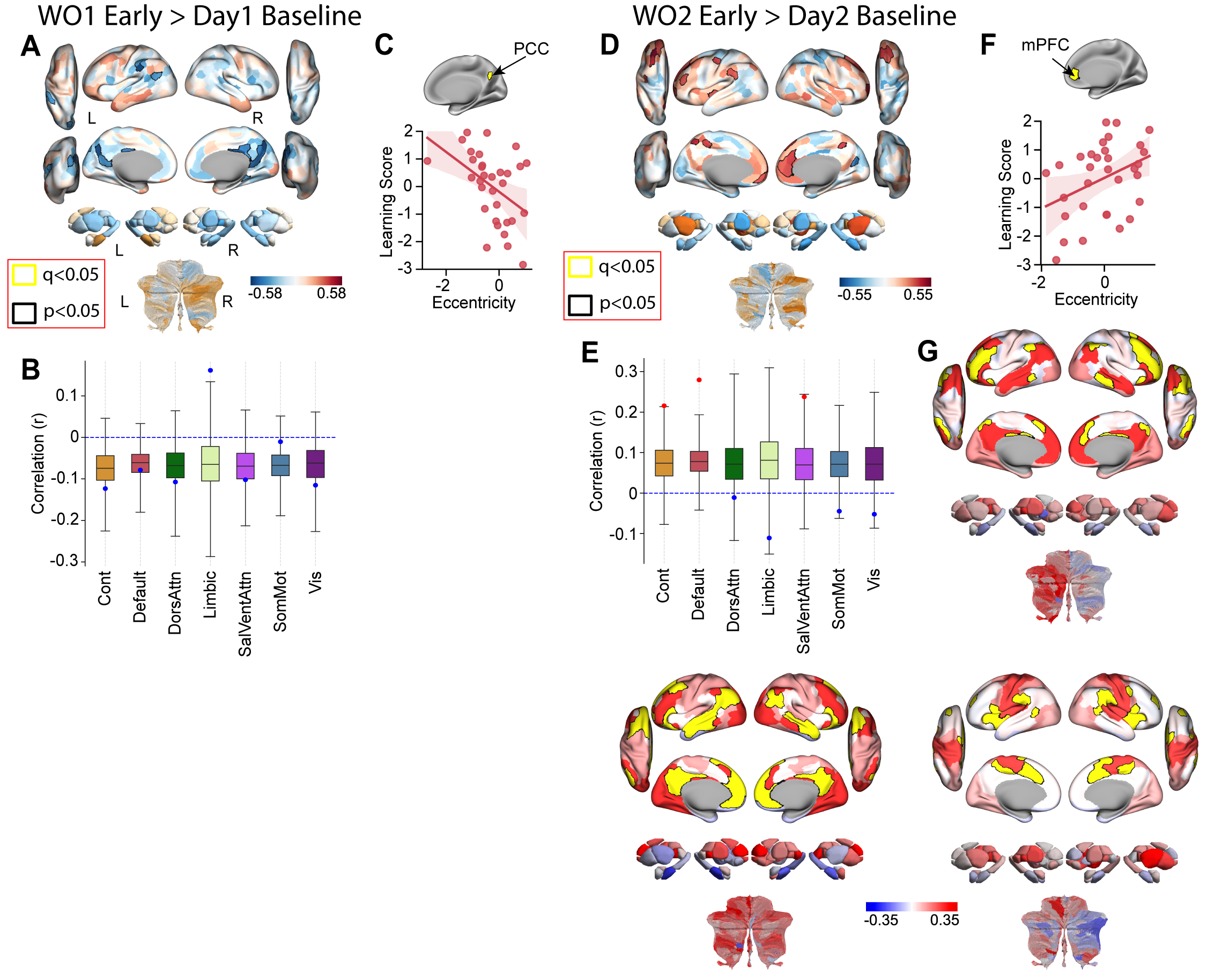

Supplement: S7 Fig — (A) Whole-brain map displaying correlations between participants’ Learning Scores and regional changes in manifold eccentricity during initial washout (WO1 Early > Day 1 Baseline). (B) Spatial permutation testing (“spin-test”) identifies functional networks whose Day 1 eccentricity changes (from A) significantly correlate with Learning Scores. Each data point shows the actual correlation for one of 7 Yeo and colleagues [1] networks. Boxplots depict the null correlation distribution (1000 iterations) for each network [2, 3]. Boxplot elements: center line = median; box edges = 25th/75th quartiles; whiskers = min-max of null distribution. Dashed blue line indicates r = 0. The data points in red denote significant network-specific correlations (FDR corrected, q < 0.05). (C) Scatterplot illustrating the correlation from panel (A) for an example brain region (indicated in yellow) with participants’ Learning Scores on Day 1. (D) Whole-brain correlation map as in (A), but for eccentricity changes during early Washout on Day 2 (WO2 Early > Day 2 Baseline). (E) Network-level spin-test results as in (B), but for the Day 2 correlations shown in (D). (F) Scatterplot illustrating the correlation from panel (E) for an example brain region on Day 2. (G) Brain maps show how the strength of connectivity change for the DMN, Control, and SalVentAttn networks correlates with the Learning Score. Red indicates that increase in connectivity with a given region is associated with better performance (higher Learning Score). Blue indicates that increased connectivity is associated with worse performance (lower Learning Score). Underlying numerical data are provided in S1 Data and archived on Zenodo (https://doi.org/10.5281/zenodo.18613054). (TIF) [file pbio.3003684.s007.tif]

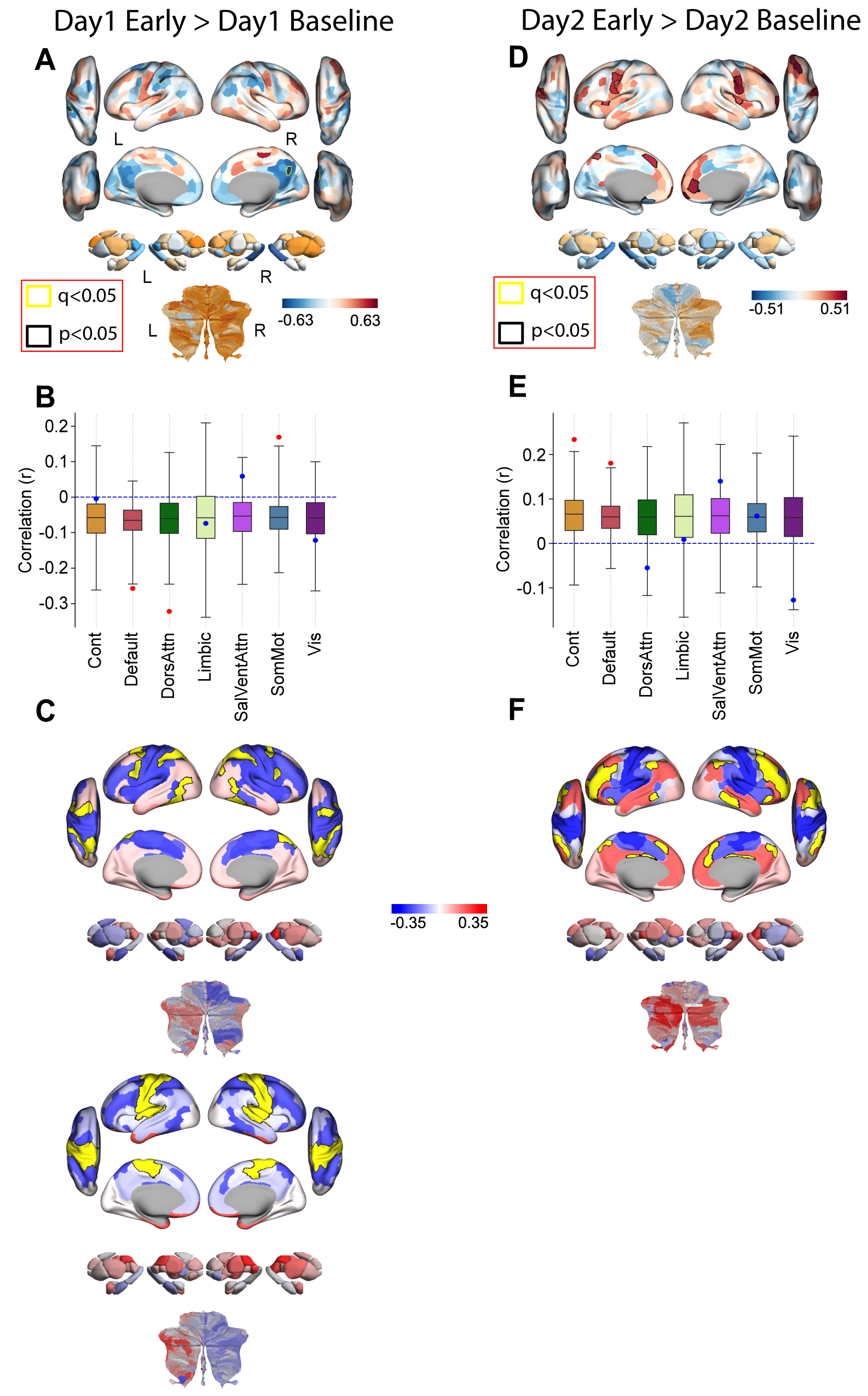

Supplement: S8 Fig — (A) Whole-brain map displaying correlations between participants’ Learning Scores and regional changes in manifold eccentricity during initial learning (Day 1 Early Learning > Day 1 Baseline). (B) Spatial permutation testing (“spin-test”) identifies functional networks whose Day 1 eccentricity changes (from A) significantly correlate with Learning Scores. Each point shows the actual correlation for one of the 7 Yeo and colleagues [1] networks. Boxplots depict the null correlation distribution (1,000 iterations) for each network [2, 3]. Boxplot elements: center line = median; box edges = 25th/75th quartiles; whiskers = min-max of null. Dashed blue line indicates r = 0. Data points in red denote significant network-specific correlations (FDR corrected, q < 0.05). (C) Brain map illustrating how Day 1 changes in functional connectivity between the DorsAttn network (top) and Somatomotor network (bottom) and all other brain regions correlate with the Learning Score. Red indicates that increased inter-network connectivity with the DorsAttn network (top) or Somatomotor network (bottom) is related to higher Learning Scores (better performance); blue indicates that increased connectivity is associated with lower Learning Scores. (D) Whole-brain correlation map as in (A), but for eccentricity changes during early relearning on Day 2 (Day 2 Early Relearning > Day 2 Baseline). (E) Network-level spin-test results as in (B), but for the Day 2 correlations shown in (D). (F) Brain map as in (C), but showing how Day 2 changes in the connectivity of the Frontoparietal Control network correlate with Learning Score. Underlying numerical data are provided in S1 Data and archived on Zenodo (https://doi.org/10.5281/zenodo.18613054). (TIF) [file pbio.3003684.s008.tif]

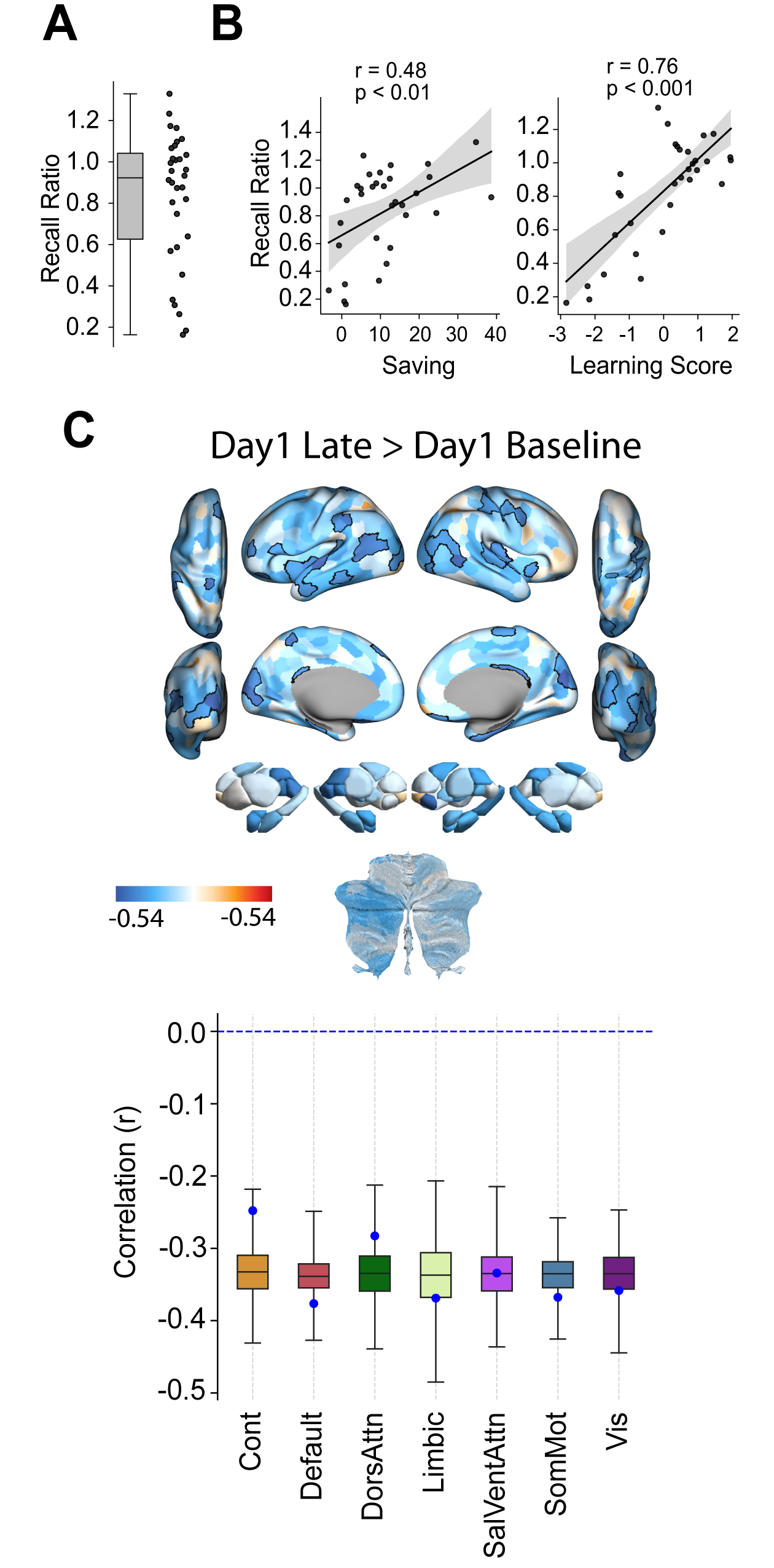

Supplement: S9 Fig — (A) Distribution of Recall Ratio (RR) values across participants, defined as (RR=AdaptationD2 Early / AdaptationD1 Late). (B) Scatterplots relating RR to behavioral savings (left) and Learning Score (right), showing significant associations with each metric (RR–Saving: r = 0.48, p < 0.01; RR–Learning Score: r = 0.76, p < 0.001). (C) Network-level correlations between RR and baseline-corrected manifold eccentricity during Day 1 Late Learning. Correlations were computed separately within each canonical functional network and assessed using the same permutation-testing framework used in Fig 7. While higher Recall Ratios were generally associated with greater manifold contraction (negative correlations) across most networks, no single network exhibited a statistically significant selective effect after permutation testing. Underlying numerical data are provided in S1 Data and archived on Zenodo (https://doi.org/10.5281/zenodo.18613054). (TIF) [file pbio.3003684.s009.tif]

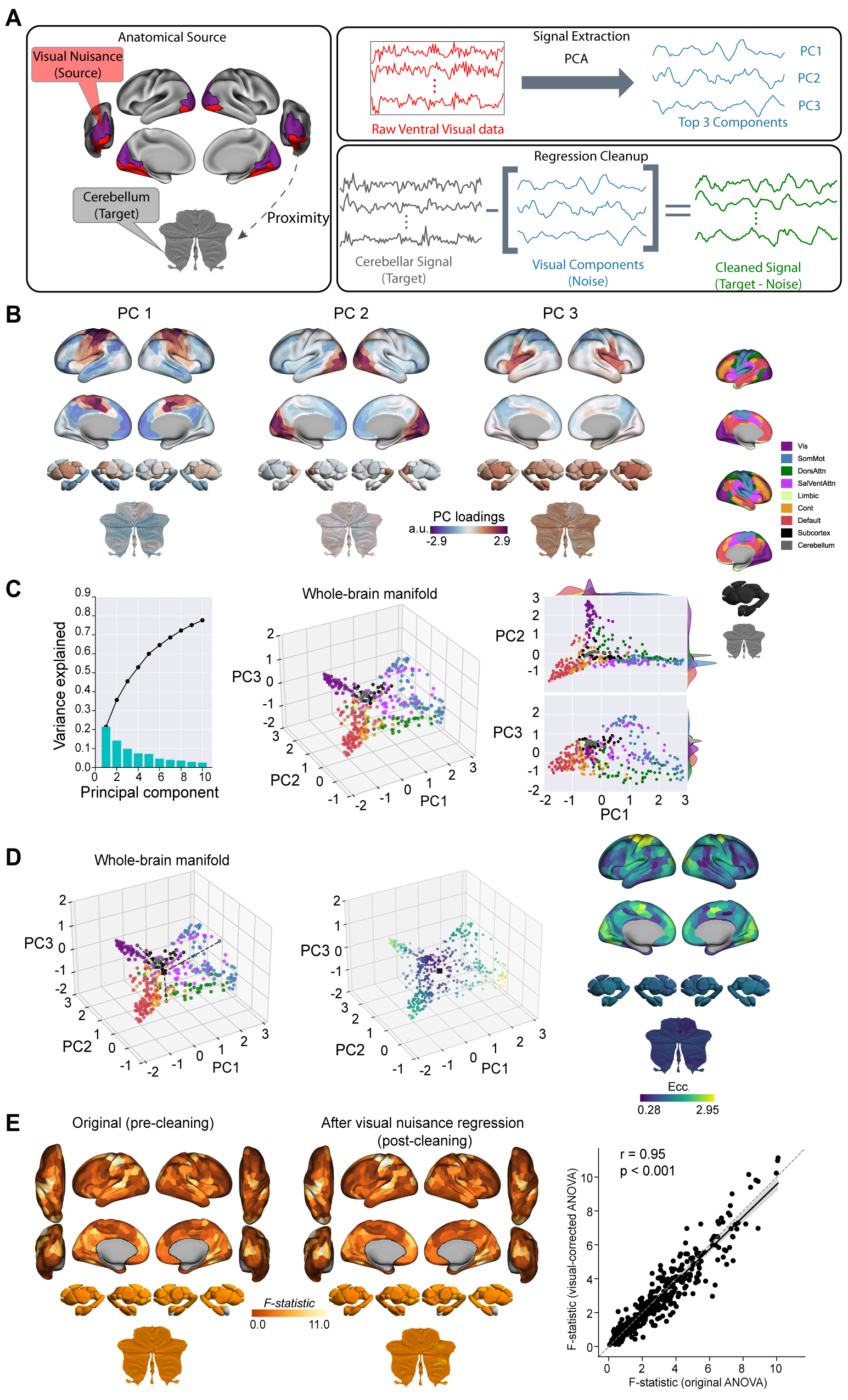

Supplement: S10 Fig — (A) Schematic of the nuisance-regression pipeline: ventral visual cortex time series were treated as a nuisance source, PCA was used to extract the top three visual components, and these components were regressed from cerebellar time series to yield a cleaned cerebellar signal. (B) Spatial loadings for the top three visual PCs for the nuisance-regressed data. (C) Whole-brain manifold structure in low-dimensional PC space for the nuisance-regressed data. (D) Manifold organization/eccentricity maps following visual-component regression. (E) Robustness of inferential results: parcel-wise F-statistics from the original ANOVA strongly correlate with F-statistics from the visual-corrected analysis (r = 0.95, p < 0.001), indicating that manifold changes are nearly identical across analysis pipelines. The dashed line is the unity line. (TIF) [file pbio.3003684.s010.tif]

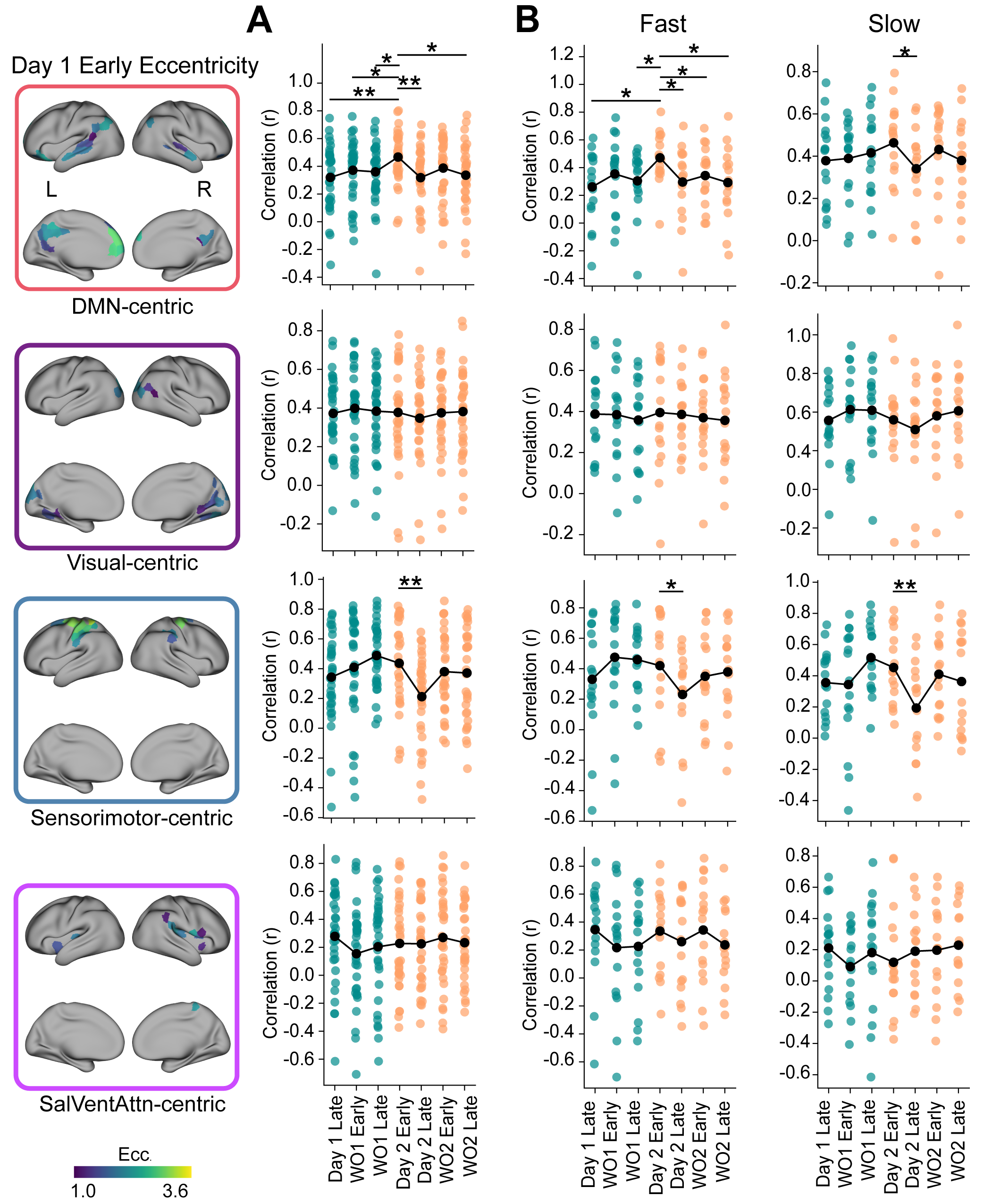

Supplement: S11 Fig — To confirm that the reinstatement effects were not driven by outliers or distributional assumptions inherent to Pearson correlation, we repeated the representational similarity analysis (RSA) using rank-based Spearman correlations. (A) The full-sample RSA results shown in Fig 4 using Spearman correlation, plotting the similarity between the Day 1 Early Learning eccentricity pattern and each subsequent learning/washout epoch separately for each functional ensemble (DMN-centric, Visual-centric, Sensorimotor-centric, SalVentAttn-centric). (B) The learner heterogeneity RSA results shown in Fig 8 using Spearman correlation, plotted separately for fast and slow learners (median split on Learning Score). In all plots, the black line shows the across-subject mean and individual points show single subjects, color-coded by day (Day 1: cyan; Day 2: orange). Asterisks denote significant effects from one-tailed paired-samples t-tests comparing the similarity between Day 1 Early Learning and Day 2 Early Relearning versus other epochs (* p < 0.05, ** p < 0.01). Across both the full sample and subgroup analyses, the key finding—a selective DMN-centric reinstatement profile with elevated similarity at Day 2 Early Relearning—remained qualitatively and statistically consistent when using Spearman correlation. Underlying numerical data are provided in S1 Data and archived on Zenodo (https://doi.org/10.5281/zenodo.18613054). (TIF) [file pbio.3003684.s011.tif]
